# Characteristics and distribution of obesity in the Arab-American population of southeastern Michigan

**DOI:** 10.1186/s12889-020-09782-3

**Published:** 2020-11-10

**Authors:** Saivaishnavi Kamatham, Joseph Trak, Suma Alzouhayli, Ziad Fehmi, Nabil Rahoui, Noor Sulieman, Zaina Khoury, Omar Fehmi, Hanan Rakine, Dana El-Masri, Deema Ujayli, Hanin Elhagehassan, James Naaman, Firas Almsaddi, Michael Salloum, Iqra Farooquee, Nadia Syed, Seongho Kim, Omar Lattouf, Michele L. Cote, Rouba Ali-Fehmi

**Affiliations:** 1 Department of Pathology, Wayne State University School of Medicine, Detroit, MI USA; 2 Wayne State University School of Medicine, Detroit, MI USA; 3 University of Michigan, Ann Arbor, MI USA; 4 Wayne State University School of Medicine, Department of Family Medicine and Public Health Sciences, Detroit, MI USA; 5 Oakland University, Rochester, MI USA; 6 Michigan State University, East Lansing, MI USA; 7 Arab Community Center for Economic and Social Services, Dearborn, MI USA; 8 Biostatistics Core, Karmanos Cancer Institute, Department of Oncology, Wayne State University School of Medicine, Detroit, MI USA; 9 Department of Surgery, Emory University School of Medicine, Atlanta, GA USA; 10 Department of Oncology, Wayne State University School of Medicine, Detroit, MI USA

**Keywords:** Arab-Americans, Body mass index (BMI), Diabetes mellitus, Employment, Hyperlipidemia, Hypertension, Michigan, Obesity

## Abstract

**Background:**

Arab-Americans constitute ~ 5% of Michigan’s population. Estimates of obesity in Arab-Americans are not up-to-date. We aim to describe the distribution of and factors associated with obesity in an Arab-American population in Southeastern Michigan (SE MI).

**Methods:**

Retrospective medical record review identified *n* = 2363 Arab-American patients seeking care at an Arab-American serving clinic in SE MI, located in a city which is home to a large proportion of Arab-Americans in the United States (US). Body mass index (BMI) was the primary outcome of interest. Distribution of BMI was described using percentages, and logistic regression models were constructed to examine the association between obesity, other comorbid conditions and health behaviors. This cohort was compared to Michigan’s Behavioral Risk Factor Surveillance System (BRFSS) data from 2018 (*n* = 9589) and to a cohort seeking care between 2013 and 2019 from a free clinic (FC) located in another city in SE MI (*n* = 1033).

**Results:**

Of the 2363 Arab-American patients, those who were older or with HTN, DM or HLD had a higher prevalence of obesity than patients who were younger or without these comorbidities (all *p*-value < 0.001). Patients with HTN were 3 times as likely to be obese than those without HTN (95% CI: 2.41–3.93; *p* < 0.001). Similarly, the odds of being obese were 2.5 times higher if the patient was diabetic (95% CI: 1.92–3.16; p < 0.001) and 2.2 times higher if the patient had HLD (95% CI: 1.75–2.83; *p* < 0.001). There was no significant difference in obesity rates between Arab-Americans (31%) and the BRFSS population (32.6%). Compared to Arab-Americans, patients seen at the FC had a higher obesity rate (52.6%; *p* < 0.001) as well as significantly higher rates of HTN, DM and HLD (all p < 0.001).

**Conclusion:**

Overall obesity rates in Arab-Americans were comparable to the population-based BRFSS rates, and lower than the patients seen at the FC. Further studies are required to understand the impact of obesity and the association of comorbidities in Arab-Americans.

## Background

Obesity rates in the United States (US) are rising at an alarming rate with adult age-adjusted prevalence of 42.4% in 2018 compared to 30.5% in the year 2000 [1]. Non-Hispanic African Americans have the highest prevalence of obesity at 49.6% compared to other ethnicities [Hispanics (44.8%), non-Hispanic whites (42.2%) and non-Hispanic Asians (17.4%)] [2]. Arab-Americans are not considered a distinct racial or ethnic group as defined by the United States Census. Hence, estimates of obesity in this population are not current or readily available. Arab-Americans experience similar environmental and behavioral conditions as the general US public; however, they represent different customs and cultural norms that may be contributing to adult and childhood obesity in their communities. The rising prevalence of obesity among genetically stable populations indicates that environmental and behavioral factors underlie the obesity epidemic [3–5]. The state of Michigan (MI) has the second-highest population of Arab-Americans in the US, with Arab-Americans residing in 82 of the 83 counties [2, 3]. Dearborn, a city located in Southeastern (SE) MI, has the largest percentage of Arab-Americans (30%) among places of similar population size in Michigan [4].

Our aim was to study the distribution and characteristics of obesity in SE Michigan’s Arab-American population. Furthermore, we compare these data to the 2018 Michigan’s Behavioral Risk Factor Surveillance System (BRFSS) [2] and to patients seeking care at a Free Clinic (FC) also located in SE MI to determine whether obesity rates were similar across different populations in MI.

## Materials and methods

### Study approval and design

The study was approved by the Wayne State University (WSU) Institutional Review Board (IRB) and the Arab Community Center for Economic and Social Services (ACCESS) Community Health and Research Center as part of an expedited review. Based on our retrospective, chart-review study design, and Paragraph 8 of the Department of Health and Human Services Code of Federal Regulations [45 CFR 46.101(b)], these institutions waived the need for obtaining consent.

### Study population

The ACCESS clinic, located in Southeast MI, is the largest Arab American community nonprofit organization in the United States, which provides public health programs that focus on the needs of Arab-Americans locally and nationwide. A retrospective analysis of Arab-American patients ages 18–98 years who received primary care at the ACCESS clinic from 2010 to 2019 was performed. Patients below 18 years and pregnant women were excluded from the study.

To further explore the distribution and characteristics of obesity in the SE MI population, we compared our cohort of Arab-American patients to two other cohorts. Firstly, the Michigan Behavioral Risk Factor Surveillance System (BRFSS) is an annual population-based telephone survey of the health behaviors of Michigan residents. Data from the 2018 report were compared to the ACCESS clinic population [6]. Next, data from individuals aged 19–64 years who sought care from 2013 to 2019 at a Free Clinic (FC) located in a different city than the ACCESS clinic in SE MI were abstracted in the same manner as were the ACCESS data. Both ACCESS and the FC offer free health care to uninsured adults in SE MI.

### Study variables

The following variables were extracted from medical records using a standardized data collection form: age, sex, marital status, employment, body mass index (BMI), history of hypertension (HTN), diabetes mellitus (DM), hyperlipidemia (HLD), tobacco use, and alcohol consumption. For abstracted data from the ACCESS clinic and FC, the following dichotomous variables were self-reported in the medical records: employment (yes = currently employed), marital status (yes = married or partnered), alcohol use (yes = ever), smoking status (yes = current; no = never or former), and hookah use (yes = current, no = never or former). The following clinical data were abstracted from the medical records, based on physician notes, vital signs, or laboratory tests: height in inches, weight in pounds, Hypertension (yes/no), Diabetes Mellitus (yes/no), Hyperlipidemia (yes/no). Body Mass Index (BMI) was calculated after converting inches into meters and pounds into kilograms, and then using the equation kg/m^2^ [7]. BMI ranges of < 18.5, 18.5 to 24.9, 25.0 to 29.9 and ≥ 30 were used to categorize patients into underweight, normal weight, overweight and obese, respectively [8]. Data from BRFSS are all self-reported and were categorized in the same manner for this analysis.

### Statistical analysis

Baseline characteristics were descriptively summarized using count and percentage for categorical variables (employment, marital status, HTN, DM, HLD, tobacco use and alcohol consumption) and median and range for continuous variables (age, height, weight and BMI). To compare baseline characteristics between two groups defined by obesity (BMI ≥30 vs. < 30), Fisher’s exact or Chi-squared tests were used for categorical variables, and Wilcoxon rank-sum test was employed for continuous variables between two groups defined by obesity (BMI ≥30 vs. < 30). Distributional comparisons of BMI and age between two cohorts of patients were performed using Chi-squared tests. Univariable and multivariable logistic regression models were fit to assess associations between ten covariates of interest (age, sex, employment, marital status, hypertension, diabetes mellitus, hyperlipidemia, alcohol use, smoking status, hookah use) and obesity (non-obese served as the reference). The subgroup logistic analyses were further performed to assess the interactions between sex and six variables of interest (hypertension, diabetes mellitus, hyperlipidemia, alcohol use, smoking status [tobacco], and hookah use) and between employment and seven variables of interest (sex, marital status, diabetes mellitus, hyperlipidemia, alcohol use, smoking status [tobacco], hookah use) on obesity. The interaction *p*-values in the subgroup analyses were adjusted for multiple comparisons using the Holm’s procedure. For the multivariable logistic analysis, the covariates were selected based on the univariable logistic regression and subgroup (interaction) analyses at an (adjusted) p-value of 0.05, resulting that four covariates (age, hypertension, diabetes mellitus and hyperlipidemia) and no interaction terms were included in the multivariable logistic model. Statistical software packages, IBM SPSS Statistics (Version 19.0) and R (Version 3.6.2) were used for all data analyses. The statistical significance was determined at alpha = 0.05.

## Results

The 2363 Arab-American patients from the ACCESS clinic had a median age of 37 years (range: 18–98), with 67.3% females (*n* = 1591) and 32.7% males (*n* = 772). The majority (30%) of Arab-Americans were in the 25–35 year age group. Based on the international BMI classification, 30% (*n* = 707) were of normal weight, 2% (*n* = 47) were underweight, 37% (*n* = 876) were overweight and 31% (*n* = 733) were obese. The age and BMI distributions of SE MI’s Arab-American population are summarized in Table 1.

**Table 1:** Baseline characteristics by obesity in ACCESS/Arab American patients

	All (N = 2363)	Obesity		***p***-value
		No (***N*** = 1630)	Yes (N = 733)	
**Age, year – median (range)**	37 (18,98)	34 (18,98)	44 (19,93)	***< 0.001***
**Age, year – no. (%)**				***< 0.001***
18–24	319 (13)	269 (17)	50 (7)	
25–35	714 (30)	562 (34)	152 (21)	
35–44	447 (19)	277 (17)	170 (23)	
45–54	391 (17)	230 (14)	161 (22)	
55–64	289 (12)	171 (10)	118 (16)	
65+	203 (9)	121 (7)	82 (11)	
**Sex – no. (%)**				0.421
Female	1591 (67)	1106 (68)	485 (66)	
Male	772 (33)	524 (32)	248 (34)	
**Height, in – median (range)**	64 (51,79)	65 (52,79)	64 (51,78)	***< 0.001***
**Weight, lbs – median (range)**	163 (79,346)	150 (79,248)	198 (117,346)	***< 0.001***
**BMI, lbs/in**^**2**^ **– median (range)**	27.43 (12.34,58.51)	25.39 (12.34,29.99)	33.47 (30.02,58.51)	***< 0.001***
**BMI, lbs/in**^**2**^ **– no. (%)**				***–***
Underweight	47 (2)	47 (3)	–	
Normal	707 (30)	707 (43)	–	
Overweight	876 (37)	876 (54)	–	
Obese	733 (31)	–	733 (100)	
**Employment – no. (%)**				0.739
No	387 (16)	275 (17)	112 (15)	
Yes	312 (13)	218 (13)	94 (13)	
Missing	1664 (70)	1137 (70)	527 (72)	
**Marital status – no. (%)**				0.266
No	119 (5)	88 (5)	31 (4)	
Yes	472 (20)	323 (20)	149 (20)	
Missing	1772 (75)	1219 (75)	553 (75)	
**Hypertension – no. (%)**				***< 0.001***
No	985 (42)	734 (45)	251 (34)	
Yes	392 (17)	191 (12)	201 (27)	
Missing	986 (42)	705 (43)	281 (38)	
**Diabetes Mellitus – no. (%)**				***< 0.001***
No	956 (40)	701 (43)	255 (35)	
Yes	370 (16)	195 (12)	175 (24)	
Missing	1037 (44)	734 (45)	303 (41)	
**Hyperlipidemia – no. (%)**				***< 0.001***
No	875 (37)	646 (40)	229 (31)	
Yes	455 (19)	254 (16)	201 (27)	
Missing	1033 (44)	730 (45)	303 (41)	
**Alcohol use – no. (%)**				0.737
No	1048 (44)	713 (44)	335 (46)	
Yes	42 (2)	30 (2)	12 (2)	
Missing	1273 (54)	887 (54)	386 (53)	
**Smoking status – no. (%)**				0.497
No	1560 (66)	1073 (66)	487 (66)	
Yes	377 (16)	252 (15)	125 (17)	
Missing	426 (18)	305 (19)	121 (17)	
**Hookah use – no. (%)**				> 0.99
No	1230 (52)	837 (51)	393 (54)	
Yes	174 (7)	118 (7)	56 (8)	
Missing	959 (41)	675 (41)	284 (39)	

The prevalence of obesity in Arab-Americans was not statistically different between men and women, 32.1 and 30.5%, respectively (*p* = 0.421, Table 1). Among Arab-Americans, patients with HTN, DM and HLD had a higher prevalence of obesity, 51.3, 47.3 and 44.2%, respectively, than patients without these comorbidities, 25.4, 26.7 and 26.2%, respectively, (all *p*-value < 0.001) as illustrated in Table 1. Obesity prevalence did not differ by alcohol use, smoking status, or hookah use.

Table 2 shows unadjusted (univariable) and adjusted (multivariable) logistic regression models estimating the association between obesity and various health conditions. The odds of being obese were 3-fold more likely among patients with HTN compared to those without HTN (OR = 3.1; 95% CI: 2.4–3.9), Similarly, the odds of being obese were 2.5-fold higher if the patient was diabetic (95% CI: 1.92–3.16; *p* < 0.001) and 2.2 times higher if the patient had HLD (95% CI: 1.75–2.83; p < 0.001), as summarized in Table 2. After adjustment for the other health conditions, only age and hypertension remained significantly associated with obesity (OR = 1.02, 95% CI: 1.01–1.03 and OR = 2.3, 95% CI: 1.5–3.3, respectively).

**Table 2:** Univariable and multivariable logistic regression analyses of factors associated with obesity (Yes vs. No, No as reference) in Arab-American patients

	Univariable			Multivariable		
	E/N	OR (95 95% CI)	***p***-value	E/N	OR (95 95% CI)	p-value
**Age**	733/2363	1.028 (1.022,1.033)	***< 0.001***	339/1130	1.016 (1.006,1.027)	***0.002***
**Sex**						
Female	485/1591	Reference				
Male	248/772	1.079 (0.896,1.298)	0.419			
**Employment**						
No	112/387	Reference				
Yes	94/312	1.059 (0.763,1.467)	0.732			
**Marital status**						
No	31/119	Reference				
Yes	149/472	1.309 (0.84,2.085)	0.243			
**Hypertension**						
No	251/985	Reference		219/899	Reference	
Yes	201/392	3.077 (2.411,3.933)	***< 0.001***	120/231	2.263 (1.533,3.348)	***< 0.001***
**Diabetes Mellitus**						
No	255/956	Reference		231/895	Reference	
Yes	175/370	2.467 (1.923,3.167)	***< 0.001***	108/235	1.048 (0.685,1.593)	0.828
**Hyperlipidemia**						
No	229/875	Reference		224/861	Reference	
Yes	201/455	2.232 (1.759,2.835)	***< 0.001***	115/269	1.129 (0.778,1.628)	0.518
**Alcohol use**						
No	335/1048	Reference				
Yes	12/42	0.851 (0.415,1.642)	0.644			
**Smoking status**						
No	487/1560	Reference				
Yes	125/377	1.093 (0.858,1.386)	0.468			
**Hookah use**						
No	393/1230	Reference				
Yes	56/174	1.011 (0.715,1.413)	0.951			

Univariable, Univariable logistic regression analysis; Multivariable, Multivariable logistic regression analysis; E/N, the number of events (i.e., obesity) and patients; OR, odds ratio; 95% CI, Confidence interval

**Fig. 1:**
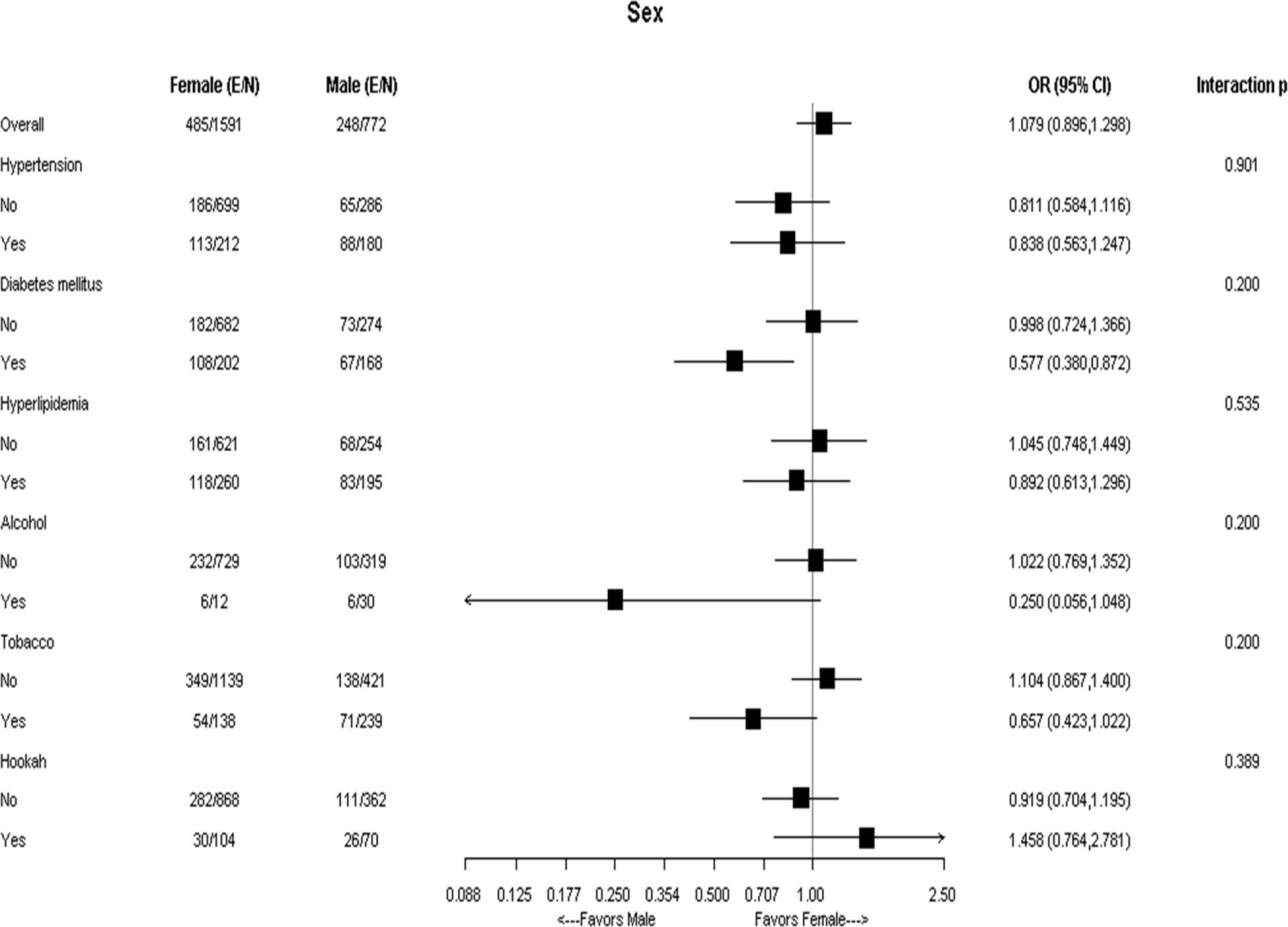
Subgroup analysis of obesity by sex in Arab-American patients (Female vs. Male). The interaction *p*-values were corrected for multiple comparisons by the Holm’s procedure

**Fig. 2:**
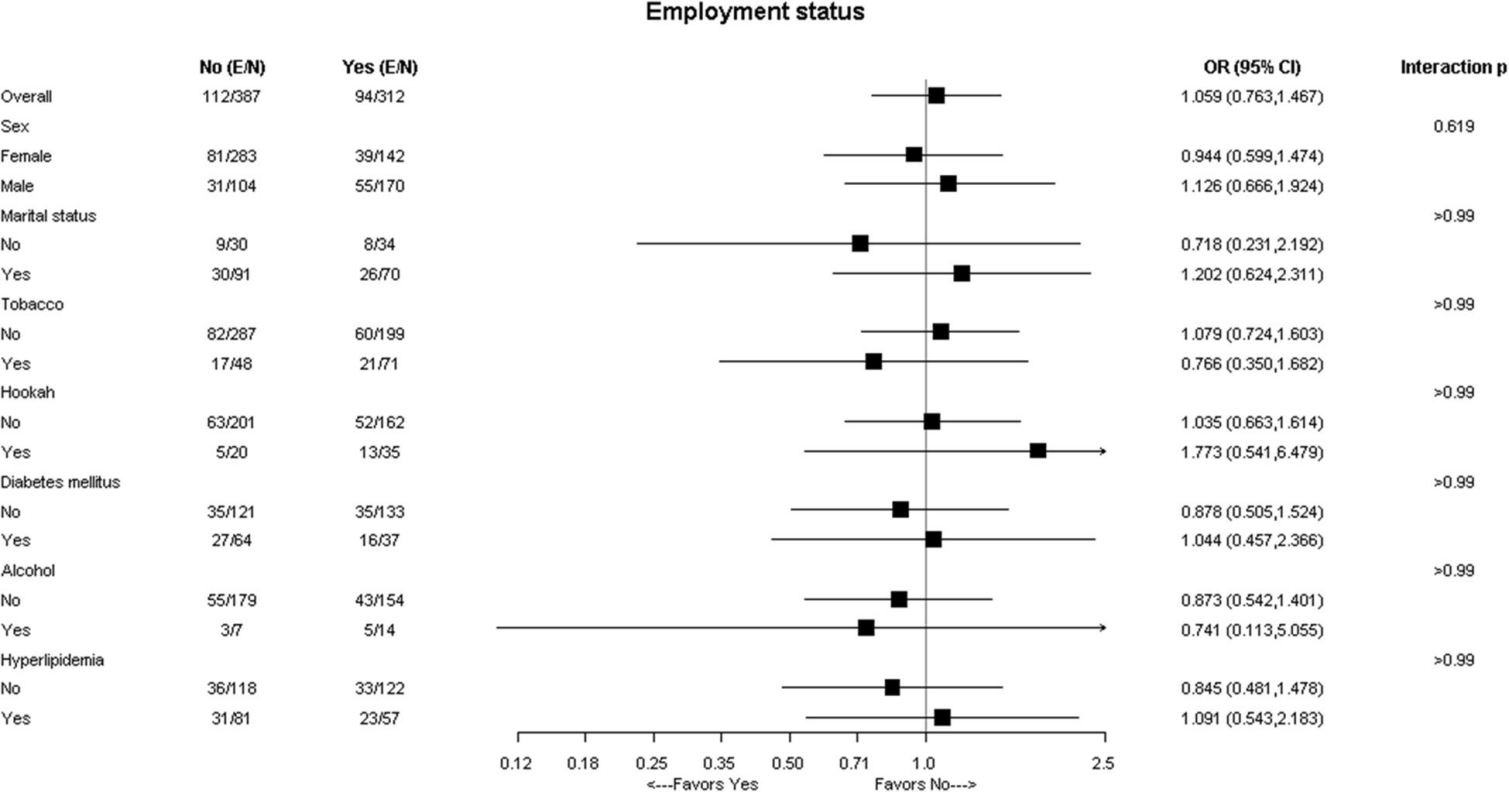
Subgroup analysis of obesity by employment status in Arab-American patients. (Yes vs. No). The interaction p-values were corrected for multiple comparisons by the Holm’s procedure

Subgroup analysis of obesity in Arab-Americans by sex and employment status are illustrated in Figs. 1 and 2, respectively. No interaction by sex or employment was detected.

Table 3 describes the distribution of BMI in the Arab-American population, the population-based, MI BRFSS data, and the FC patient population. No difference in distribution is noted between the Arab-American and BRFSS population (*p* = 0.278), but the Arab-American and FC populations were significantly different (*p* < 0.001), with the FC population more likely to be obese.

**Table 3:** Comparison of BMI distribution among Arab-American/ACCESS, MI-BRFSS and FernCare Free Clinic data

	Arab-Americans/ACCESS	MI-BRFSS	FCFC patients	p-value^*^	
				vs. MI-BRFSS	vs. FCFC
**BMI – no. (%)**				0.278	***< 0.001***
Underweight	47 (2)	157 (2)	5 (1)		
Normal	707 (30)	2879 (30)	201 (19)		
Overweight	876 (37)	3425 (35)	284 (27)		
Obese	733 (31)	3128 (33)	543 (53)		
Total	2363 (100)	9589 (100)	1033 (100)		

^*^ Pair-wise Chi-squared p-values between ACCESS and BRFSS and between ACCESS and FC Free Clinic

## Discussion

In this study, we described the distribution and characteristics of obesity in three different groups: Arab-Americans of SE MI, data from the Michigan BRFSS, and individuals seeking care from the FC. Our results demonstrated the following: (i) the prevalence of obesity in Arab-Americans did not differ by sex, (ii) HTN, DM and HLD were associated with obesity in the Arab-American population, (iii) there is no significant difference in obesity rates between Arab-Americans and MI’s BRFSS population, (iv) the rates of obesity in the patients from the FC were higher than those in the Arab-American population. The similarities between this Arab American population and other racial and ethnic groups in MI suggests that interventions to promote healthier behaviors (e.g. to lower the prevalence of obesity) are also needed in the Arab-American community.

We report no difference in obesity rates by sex in the Arab-American population. The similar prevalence of obesity by sex is also seen in non-Hispanic white, Hispanic, and Asian populations, but differs from what has been shown in the African American population, where women are more likely to be obese [2]. The obesity rates are similar to population-based estimates in MI (e.g. BRFSS) as well as rates reported for various countries of origin, including Iraq, Lebanon, and Saudi Arabia [9]. We did not collection information on variables to measure acculturation, such as length of time in the US or primary language spoken in the home; however, the primary population that the ACCESS clinic caters to is comprised of first-generation individuals who are still following the dietary customs of their countries of origin. A study of third-generation Arab-Americans in California reported that they were 2.59 times and 3.22 times more likely to be overweight or obese compared to first- and second-generation Arab-Americans, respectively. Furthermore, their results also revealed a higher likelihood of binge drinking in second-generation California-based Arab-Americans compared to first-generation Arab-Americans (adjusted odds ratio [AOR] = 3.26; 95% CI:1.53–6.94) [10]. It is possible obesity prevalence in our population may also differ based on generation of entry into the US.

Based on our results, the association of obesity in our study population and several comorbidities including hypertension, diabetes mellitus, and hyperlipidemia are in accordance with previous studies [11–13]. Baik et al., reported the association of obesity with overall and cause-specific mortality in US men aged 40–75 years. Their results showed that the risk of cardiovascular disease mortality among men aged < 65 years increased linearly with higher BMI [14]. In 2016, Hruby et al. used data from the Nurses’ Health Study to show that overweightness and obesity are important risk factors for diabetes mellitus, cardiovascular diseases, cancers and early death among women [15]. Interventions to reduce body weight, increase activity, and other measures that can help achieve and maintain a healthy weight status are critical to controlling the obesity epidemic. A study of 267 Arab-American women in California found very low rates of self-reported physical activity, suggesting that this area of health promotion may benefit this population in particular [16]. Another systematic review suggests that knowledge of healthy behaviors, including the benefits of physical activity is increasing in Arab-Americans, but longitudinal data are still lacking, as is the methodology to capture the diversity within the Arab-American population [17].

The prevalence of obesity among those seeking care at the FC was higher than that of Arab-Americans and the MI-BRFSS population. This may be due to socioeconomic status and several other factors. The impact of socioeconomic factors such as employment on obesity was well described by Levine et al. who reported that individuals living in underresourced regions have diminished access to fresh food and were more susceptible to a sedentary lifestyle [18]. In an systematic literature review from 1996 to 2011 on sedentary behaviors and subsequent health outcomes in adults by Thorp et al., a consistent association of self-reported sedentary behavior with obesity from childhood to adulthood was reported [19]. Żukiewicz-Sobczak et al. described the association between obesity and low socioeconomic status in developed countries such as the United States and United Kingdom. In their study, they described higher levels of unemployment, lower education levels, irregular meal patterns and reduced physical activity among the lower socioeconomic sector as the main reasons for obesity in the underprivileged [20]. It is possible that the Arab-American population in our study, despite seeking care at a no-cost clinic, has better resources compared to individuals seeing care at the no-cost FC.

Our study has several strengths. First, the large Arab-American sample size (*n* = 2363) across a broad age range provides obesity estimates across a wide age range. Second, we were able to examine the association between obesity and other comorbid conditions. Third, we were able to compare this Arab-American population with Michigan’s population-based BRFSS data and with data from the FC clinic.

Our study was not without limitations. First, the retrospective nature of the study and lack of complete data found in the patient’s paper charts. Second, the inability to eliminate the possibility that data quality or availability differed by obesity status (for example, more tests were performed on obese patients compared to non-obese patients) may affect accuracy of the associations between obesity and the various studied factors. Third, we were unable to examine factors such as lower education status or type/quality of employment, that may have an effect on socioeconomic status and influence obesity. Given the various unfavorable outcomes of obesity, it would be beneficial to study the aforementioned limitations in a prospective study.

## Conclusion

We described the charecteristics and distribution of obesity in Arab-Americans of SE MI and have found similar prevalence compared to population-based data from the State of Michigan. Given that over two-thirds of the Arab-American population studied is overweight or obese, a focus on interventions to decrease obesity, through culturally-approprate methods, is warranted.

## Data Availability

All data generated or analysed during this study are included in this published article. Data for the population of Michigan was acquired fom BRFSS published data 2018. https://nccd.cdc.gov/BRFSSPrevalence/rdPage.aspx?rdReport=DPH_BRFSS.ExploreByLocation&rdProcessAction=&SaveFileGenerated=1&irbLocationType=States&islLocation=26&islState=&islCounty=&islClass=CLASS14&islTopic=TOPIC09&islYear=2018&hidLocationType=States&hidLocation=26&hidClass=CLASS14&hidTopic=TOPIC09&hidTopicName=BMI+Categories&hidYear=2018&irbShowFootnotes=Show&rdICL-iclIndicators=_BMI5CAT&iclIndicators_rdExpandedCollapsedHistory=&iclIndicators=_BMI5CAT&hidPreviouslySelectedIndicators=&DashboardColumnCount=2&rdShowElementHistory=&rdScrollX=0&rdScrollY=0&rdRnd=88232

## Abbreviations

MI: Michigan
BRFSS: Behavioral Risk Factor Surveillance System
FC: FC Free Clinic
ACCESS: Arab Community Center for Economic and Social Services
BMI: Body Mass Index
HTN: Hypertension
DM: Diabetes Mellitus
HLD: Hyperlipidemia
US: United States
CDC: Centers for Disease Control and Prevention
SE: Southeastern
